# A Loss of Nuclear—Cytoskeletal Interactions in Vascular Smooth Muscle Cell Differentiation Induced by a Micro-Grooved Collagen Substrate Enabling the Modeling of an In Vivo Cell Arrangement

**DOI:** 10.3390/bioengineering8090124

**Published:** 2021-09-12

**Authors:** Kazuaki Nagayama

**Affiliations:** Micro-Nano Biomechanics Laboratory, Department of Mechanical Systems Engineering, Ibaraki University, Nakanarusawa-cho, Hitachi 316-8511, Japan; kazuaki.nagayama.bio@vc.ibaraki.ac.jp

**Keywords:** cell biomechanics, mechanobiology, vascular smooth muscle cell, cytoskeleton, nucleus

## Abstract

Vascular smooth muscle cells (VSMCs) remodel vascular walls actively owing to mechanical cues and dedifferentiate to the synthetic phenotype from contractile phenotype in pathological conditions. It is crucial to clarify the mechanisms behind the VSMC phenotypic transition for elucidating their role in the vascular adaptation and repair and for designing engineered tissues. We recently developed novel micro-grooved collagen substrates with “wavy wrinkle” grooves to induce cell–substrate adhesion, morphological polarization, and a tissue-like cell arrangement with cytoskeletal rearrangements similar to those in vascular tissue in vivo. We found that cultivation with this micro-grooved collagen significantly induced VSMC contractile differentiation. Nonetheless, the detailed mechanism underlying the promotion of such VSMC differentiation by micro-grooved collagen has not been clarified yet. Here, we investigated the detailed mechanism of the cell arrangement into a tissue and contractile-differentiation improvement by our micro-grooved collagen substrates in terms of nuclear–cytoskeletal interactions that possibly affect the nuclear mechanotransduction involved in the activation of transcription factors. We found that VSMCs on micro-grooved collagen manifested significant cell arrangement into a tissue and nucleus slimming with a volume reduction in response to the remodeling of the actin cytoskeleton, with consequent inhibition of nuclear shuttling of a transcriptional coactivator, Yes-associated protein (YAP), and improved contractile differentiation. Furthermore, VSMC nuclei rarely deformed during macroscopic cell stretching and featured a loss of nesprin-1–mediated nuclear–cytoskeletal interactions. These results indicate that our micro-grooved collagen induces a cell alignment mimicking in vivo VSMC tissue and promotes contractile differentiation. In such processes of contractile differentiation, mechanical interaction between the nucleus and actin cytoskeleton may diminish to prevent a nuclear disturbance from the excess mechanical stress that might be essential for maintaining vascular functions.

## 1. Introduction

Vascular smooth muscle cells (VSMCs) constitute the main components of the arterial wall, and these regulate contraction and dilation of the walls as well as vigorously remodel the arterial wall in which they reside via biochemical cues from endothelial cells and mechanical signals that are self-applied [1,2,3], and keep mechanical stress in the wall at a normal level via their contractility [3]. In healthy vascular walls, differentiated mature VSMCs possess a contractile phenotype. Contractile VSMCs feature elongated bipolar morphology and stay quiescent in proliferation and metabolism of the ECM (extracellular matrix) [4]. In pathologies, such as hypertension and atherogenesis, VSMCs go through dedifferentiation to the synthetic phenotype from the contractile phenotype. Synthetic VSMCs express fewer contractile proteins, possess a stellate shape, and are operating during migration, proliferation, and ECM turnover [5]. Similar cellular dedifferentiation is seen when VSMCs are placed in culture conditions after removal from a native tissue; VSMCs randomly spread on the flat surface in a culture dish and show a less elongated morphology. The mechanism behind VSMC differentiation and dedifferentiation is crucial for vascular repair and adaptation, and for the pathophysiology of some diseases. Besides, elucidation of differentiation/dedifferentiation mechanisms of VSMCs can help to design tissue-engineered blood vessels.

From a biomechanical viewpoint, influences of mechanical characteristics of cell culture substrates have been in the spotlight of recent studies. Previous studies have addressed cellular responses to substrate stiffness [6,7,8] and have revealed that protein and mRNA expression levels of contractile proteins significantly increase in cells cultured on substrates having moderate surface stiffness. Microenvironments of substrate surfaces are also important for cellular functions. Microfabricated and micropatterned substrates produced by micro-electro-mechanical system (MEMS) techniques, e.g., photolithography, have been employed as powerful tools for controlling cell spreading and morphology through the creation of well-defined surface structures [9]. Specifically, adhesive micropatterns emulating bipolar-cell elongated shapes seen in vivo [10] and those featuring microfabricated grooves with several micrometer-patterned widths [11,12,13] induce cytoskeletal remodeling and cell elongation and influence cell adhesion, migration, and differentiation. On the other hand, in general, this technique may be costly and time-consuming for the production of a mold needed to fabricate the substrates. Furthermore, relatively hard (over ~2 MPa) surfaces of MEMS-based microfabricated substrates typically require certain hydrophilic processing with subsequent coating by cell adhesion proteins, e.g., collagen or fibronectin. If this protein coating constitutes a very thin layer (a few micrometers or thinner), then cells also detect mechanical properties of the hard underlying substrate that have an effect on cellular responses, whereas a thick protein layer makes the substrate surface micropatterns vague.

To overcome these issues, we recently devised a cell culture micro-grooved substrate consisting of only a type I collagen matrix that offers a substantial basal level of adhesivity by means of RGD peptide sequences, to bring about substrate–cell adhesion, elongation of cells, and a tissue-like cell arrangement with cytoskeletal rearrangements similar to those in vascular tissue in vivo [14]. We found that cultivation with this micro-grooved collagen significantly induces contractile differentiation of rat vascular smooth muscle cell lines. Nevertheless, the detailed mechanism of such VSMC differentiation facilitated by micro-grooved collagen has not been described yet.

Cells on a substrate generate internal forces through contraction of the actin–myosin cytoskeletons. These forces are crucial for cellular mechanotransduction and are potentially affected by substrate geometry. The forces are transmitted from the cytoskeleton to the nucleus via nuclear-membrane proteins that are constituents of a LINC (linker of nucleoskeleton and cytoskeleton) complex [15], which possibly affects the mechanical environment of the nucleus and transcription factors [16]. Previously, we found that actin stress fibers in VSMCs cultured on the flat surface of a dish are firmly connected to the nucleus, and the internal forces of stress fibers are transmitted directly to the nucleus [17]. Nonetheless, these nuclear–cytoskeletal interactions were proved only in synthetic dedifferentiated cell lines with a less elongated morphology. Possibly, the nuclear–cytoskeletal interactions and their efficiency of force transmission are associated with the cell differentiation induced by the substrate geometry.

Thus, in this study, we used primary porcine VSMCs to investigate the detailed mechanism of the cell arrangement into a tissue and contractile-differentiation improvement by our originally designed micro-grooved collagen substrate in terms of nuclear mechanics and nuclear–cytoskeletal interactions. Incidentally, a Yes-associated protein (YAP), Hippo effector, and transcriptional coactivator with PDZ-binding motif (TAZ) serve as a mechanosensing on–off switch that regulates multiple cellular processes. YAP and TAZ localize into the nucleus and become transcriptionally active in the cell in response to mechanical cues such as substrate stiffening [18] and mechanical stretch [19]. Furthermore, YAP has been suggested to play the important role of a mediator in the phenotypic transition of VSMCs and vascular remodeling under mechanical stretching [20,21]. Therefore, we also examined changes in intracellular localization of YAP signals during VSMC cultivation on the micro-grooved collagen. Finally, we measured the deformation of the nucleus during macroscopic cell stretch to model in vivo vascular stretching and assess the influence of the tissue-like VSMC arrangement induced by the micro-grooved collagen substrate on intracellular force transmission efficiency.

## 2. Materials and Methods

### 2.1. Fabrication of the Micro-Grooved Collagen Substrate

**Figure 1:**
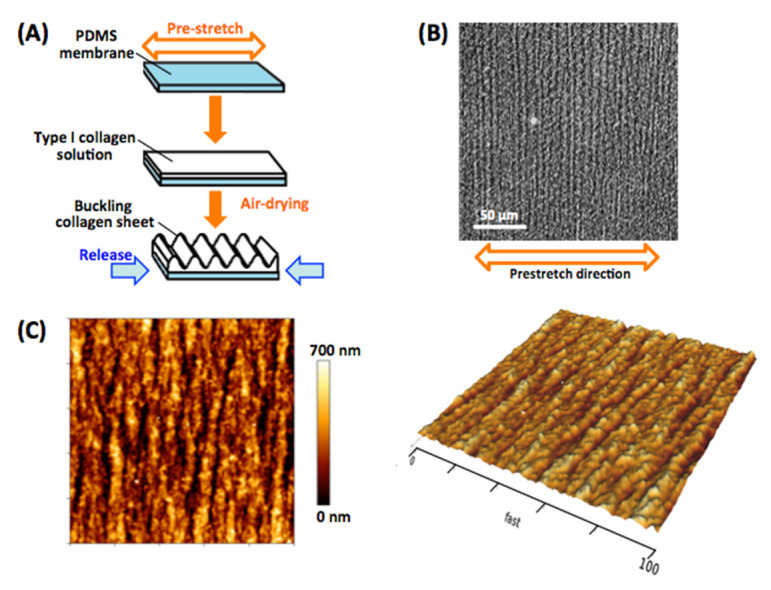
Process flowchart for fabricating the micro-grooved collagen substrate (**A**). A bright-field image of the micro-grooved collagen substrate (**B**). Typical atomic force microscopy (AFM) images of the surface topography of the micro-grooved collagen substrate samples (**C**). Precisely controlled, high-speed AFM indentation tests were performed to construct a force–displacement curve for each pixel of a 128 × 128 pixels area.

The micro-grooved collagen substrates were fabricated as described previously [14]. Briefly, a silicone rubber membrane made from polydimethylsiloxane (PDMS) (Sylgard 184, Dow-Corning) was prestretched at strain values of 25%, then a drop of a type I collagen solution (AteloCell I, AC-50, native collagen from the bovine dermis, KOKEN) was placed onto the PDMS membrane and air-dried completely at room temperature (25 °C) for 2 days (Figure 1A). The membrane was unloaded next, which caused a buckling of collagen matrix (Figure 1B). Mechanical properties and surface topography of samples of the micro-grooved collagen substrates were determined by atomic force microscopy (AFM) imaging (Figure 1C). The collagen micro-grooved substrates featured branched grooves resembling “wavy wrinkles,” with Rz = 580 ± 220 nm, Ra = 130 ± 85 nm, and groove width 5.3 ± 2.4 µm (n = 10 substrates). The average elastic modulus was 370 ± 160 kPa for the micro-grooved collagen substrate (n = 20 substrates).

### 2.2. AFM Analyses of Substrate Surface Topography and Stiffness

To determine the surface topography and elastic modulus of the collagen substrates, AFM analyses were carried out by means of a NanoWizard IV AFM instrument (JPK Instruments-AG, Berlin, Germany) mounted on top of an inverted optical microscope (IX73, Olympus, Tokyo, Japan) equipped with a digital CMOS (complementary metal oxide semiconductor) camera (Zyla, Andor). The substrates were immersed at room temperature in phosphate-buffered saline [PBS(-)]. AFM quantitative imaging (QI) mode was then applied to construct a force–displacement curve for each pixel of a measured area of 128 × 128 pixels (100 × 100 µm) obtained by a precisely controlled high-speed indentation test. We used rectangular silicon nitride cantilevers equipped with a cone probe tip (BioLever-mini, BL-AC40TS-C2, Olympus, Japan) with a nominal tip radius of 10 nm and a spring constant of 0.06–0.08 N/m. These high-speed indentations were carried out until a force was reached at a preset force of 1.5 nN. Substrate elasticity was computed from the obtained force–displacement curves via application of the Hertzian model [22], which approximates a sample as linearly elastic and isotropic. Elastic (Young’s) modulus was determined through fitting of all the force–displacement curves to the following approximation of a Hertzian model:

(1) $$F=\frac{2E\cdot \tan\alpha }{\pi \left(1-\nu^{2}\right)}\delta^{2}$$

where *E* is the elastic modulus, *F* is the applied force, *α* is an opening angle of the cone of the cantilever tip, *ν* is the Poisson ratio (for a noncompressible biological sample, it is 0.5), and *δ* is indentation depth of the sample registered in the force–displacement curves. From the results of the Hertzian model approximation, we identified elastic modulus and Z contact points (specimen surface) of the substrates in each pixel and generated a surface topography map and an elastic modulus map of the substrates.

### 2.3. Cell Culture

Porcine aortic VSMCs served as the test model. Primary VSMCs were prepared by the explant method described elsewhere [23]. They were cultivated in a standard culture proliferative medium, which consisted of Dulbecco’s modified Eagle’s medium (DMEM, Wako) supplemented with 10% of fetal bovine serum (FBS, JRH Bioscience, Lenexa, KS, USA), penicillin (100 U/mL), and streptomycin (100 μg/mL) (Sigma-Aldrich, St. Louis, MO, USA) at 37 °C and 5% CO_2_. The cells were subcultured repeatedly in a 1:4 split ratio when they reached approximately 80% confluence. Primary VSMCs at passages 6–8 were employed in all the experiments. VSMCs were seeded onto either the flat collagen or micro-grooved collagen substrate. Initial cell density was maintained at a relatively high value of ~200 cells/mm^2^ since low initial cell density (for example, 50 cells/mm^2^) decreased cell–cell contact guidance, which also inhibited tissue formation from aligned cells. The VSMCs cultured on either substrate for 3 days that gave rise to confluent tissues were subjected to subsequent experiments.

### 2.4. The Immunofluorescence Assay

The VSMCs cultured on either the micro-grooved or flat collagen substrate were fixed for 10 min with PBS(-) (Nissui, Tokyo, Japan) supplemented with 3.7% of formaldehyde, were permeabilized for 5 min with PBS supplemented with 0.5% of Triton X-100 (ICN Biomedicals, Irvine, CA, USA), and were rinsed with PBS supplemented with 1% of bovine serum albumin (BSA) to block nonspecific binding of proteins. The fixed cells were incubated for 30 min with this blocking solution before treatment with staining reagents. The major protein markers of smooth-muscle contractile differentiation—α-SMA, calponin, and transgelin—or nesprin 1 (a component of the LINC complex) were stained fluorescently as follows: The samples were probed with a mouse antibody against α-SMA (1:200 dilution, cat. # A2547, clone 1A4, Sigma), mouse antibody against calponin (1:200 dilution, C2687, Sigma), mouse antibody against transgelin (1:200 dilution, ab14106, Abcam, Cambridge, UK), or rabbit antibody against nesprin 1 (1:200 dilution, ab24742, Abcam) overnight at 4 °C. This was followed by 1 h incubation at room temperature with a secondary antibody (a rabbit anti-mouse IgG antibody conjugated with Alexa Fluor 488; 1:200 dilution, Invitrogen) for α-SMA, calponin, and transgelin or with a goat anti-rabbit IgG antibody conjugated with Alexa Fluor 488 (1:200 dilution, Invitrogen) for nesprin 1. All the antibodies were diluted with PBS(-) containing 1% of BSA. Additionally, actin filaments inside cells were fluorescently stained by incubation for 60 min with Alexa Fluor 546–conjugated phalloidin (~200 nM; Molecular Probes, Eugene, OR, USA). For fluorescent visualization of nuclei, we stained intranuclear DNA for 30 min with Hoechst 33342 (Invitrogen, Carlsbad, CA, USA).

### 2.5. Confocal Fluorescence Microscopy

Stacked fluorescent cross-sectional images of the stained VSMCs were captured by means of a confocal fluorescence microscope in the range of cell thickness (~15 µm) with 0.5 µm intervals by means of a 60× oil immersion objective lens (with numerical aperture of 1.3) or a 40× objective lens (numerical aperture = 0.95), a confocal system (CSU-X1; Yokogawa, Tokyo, Japan) with a multicolor fluorescence system (Light Engine Spectra-X; Opto-line, Osaka, Japan), and a CMOS digital camera (ORCA-Flash4.0 V2; Hamamatsu Photonics, Hamamatsu City, Japan). A nucleus outline was traced automatically, and the projected area, length, width, and volume of the nucleus were determined in the ImageJ software (NIH, Bethesda, MD, USA). The obtained stacked cross-sectional images of the cells were also transformed into 2D images of superimposed projection to evaluate fluorescence intensity of the intranuclear DNA and contractile proteins [24].

### 2.6. The EdU Cell Proliferation Assay

EdU is a nucleoside analogue of thymidine and is incorporated into DNA during only DNA synthesis, thus enabling the visualization of newly synthesized DNA [25]. This technique is a less toxic alternative to a BrdU incorporation assay. VSMCs that were seeded on either the flat or micro-grooved collagen substrate were incubated for 24 h at 37 °C and treated with EdU for 6 h in a special buffer (Click iT EdU Imaging Kits, Molecular Probes) at 37 °C, according to the manufacturer’s instructions, to ensure capture of most of proliferating cells. After the EdU treatment for 6 h, the cells were fixed, permeabilized, blocked to preclude nonspecific binding of proteins, and were stained with Alexa Fluor 488 for another 30 min in Cu(I)-catalyzed click reaction conditions, as recommended by the manufacturer. The cells were rinsed with PBS(-) and stained fluorescently for their intranuclear DNA and F-actin cytoskeleton, as detailed in Section 2.4.

### 2.7. AFM Indentation Analysis of the Cell Nucleus

For a comparison of mechanical properties of nuclei, force–indentation responses of a nucleus were determined by AFM as described in Section 2.2. For the AFM measurements, VSMCs on either the flat or micro-grooved collagen substrate were precultured for 3 days, and then their intranuclear DNA was stained with Hoechst 33342. In a preliminary study, we quantitatively compared mechanical parameters of nuclear regions of cells with F-actin disruption, of intact cells, and isolated nuclei obtained by cell lysis [26]. Elastic modulus of the nuclear region was twofold higher in the intact cells in comparison with the F-actin disruption group of cells, suggesting that nuclear stiffness is negatively affected by F-actin structures. There was no significant difference in elastic modulus between isolated nuclei and the nuclear region in the cells with F-actin disruption. Accordingly, actin filaments inside cells were depolymerized by treatment with cytochalasin D (2 µg/mL, 1 h) immediately before indentation analyses to preclude the mechanical influences of actin filaments or cell lysis. For the indentation measurements during AFM, a pyramidal tip of V-shaped silicon nitride cantilevers (MSNL-10, Bruker AXS) was used at a spring constant of 0.07 N/m and a nominal tip radius of 20 nm. The tip of a cantilever was placed over the middle region of a nucleus, as determined by optical-microscopy monitoring. Indentations were carried out at five points in the middle region of each nucleus at a constant indentation speed of 500 nm/s until a force was reached at a preset force of 1 nN. Typically, this force corresponded to 2–3 µm nuclear indentation depths. All the measurements were done within 1 h of the transfer to the AFM instrument. Nuclear elasticity values were determined via the force–indentation curves through application of the Hertzian model, as detailed in Section 2.2.

### 2.8. In Situ Measurement of Nucleus Deformation during Stretching

Either the micro-grooved or flat collagen substrate on the PDMS membrane described in Section 2.1 was glued to the bottom of rectangular stretch chambers made of silicone rubber (STB-CH-04, STREX, Osaka, Japan). VSMCs were then cultured on the collagen substrates in the stretch chambers, precultured for 3 days, and then placed on a homemade stretching apparatus with a CO_2_ incubator [27]. Before cell stretching, the cell membrane and nucleus were stained fluorescently with the Cell mask™ Green Plasma Membrane Stain Kit (C37608, Invitrogen) and Hoechst 33342, respectively. Next, the stained cells were stretched through stretching of the substrate incrementally by 5% every 20 s until the substrate strain reached 25%. Fluorescent images of the cell membrane and nucleus were captured by a C9100-12 electron-multiplying CCD (charge-coupled device) camera (Hamamatsu Photonics, Hamamatsu, Japan) connected to the microscope during the stretching. The cells whose initial major axis direction was within 20° of the stretching direction were selected as the target cells, and the elongation of the cell body and nucleus was measured manually by tracing the outline of the cells and nuclei in each image, respectively.

## 3. Results

### 3.1. Significant Alignment and Morphological Changes of VSMCs on the Micro-Grooved Collagen

**Figure 2:**
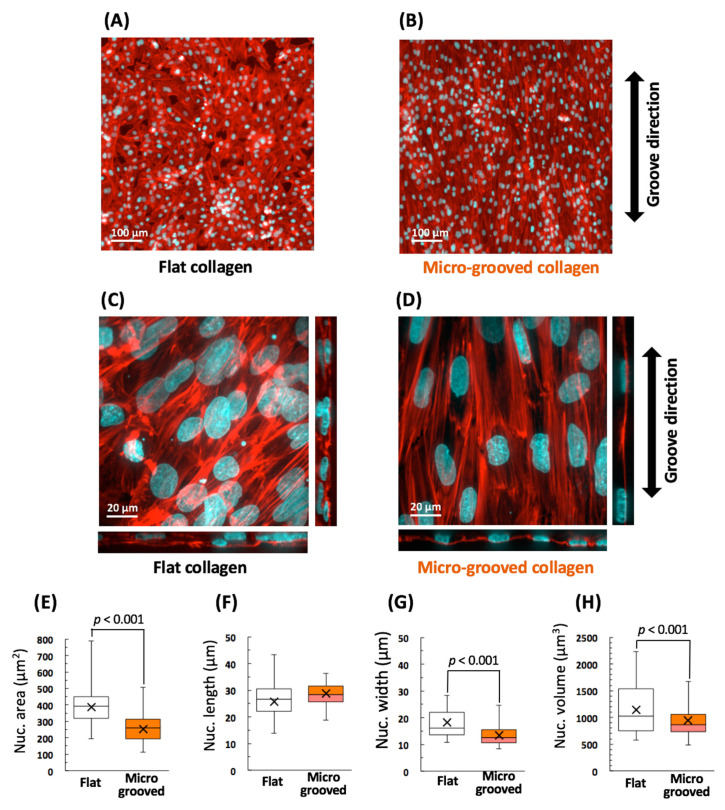
Typical fluorescent images of vascular smooth muscle cells (VSMCs) cultured on either the flat (**A**) or micro-grooved (**B**) collagen (col) substrate. Actin filaments and nuclei were visualized using Alexa Fluor–conjugated phalloidin (red) and Hoechst 33342 (cyan), respectively. Examples of confocal fluorescent images of VSMCs cultured on either the flat (**C**) or micro-grooved (**D**) collagen substrate for 3 days. The obtained stacked cross-sectional images of the cells were converted to superimposed projection 2D images and their reconstructed lateral images. Shapes of the nuclei were quantified via these confocal images (**E**–**H**). Over 500 cells were analyzed in each group. (Flat, n = 505 cells; Micro-grooved, n = 530 cells).

First, we examined the effects of surface topography of the micro-grooved collagen substrate on cell alignment and intracellular structures, especially, the actin cytoskeleton and nucleus. Confocal fluorescent imaging revealed that VSMCs cultured on the conventional flat collagen substrate possessed irregular shapes with actin fibers of random orientation, and nucleus orientation differed among the cells (Figure 2A,C). Some cells formed multiple layers by spreading on other cells (Figure 2C). In contrast, the VSMCs cultivated on the collagen micro-grooved substrate elongated in the grooves’ direction and yielded an aligned cell arrangement (Figure 2B,D) similar to a tissue seen in vivo. The cells tended to form a single-cell layer (Figure 2D). Both the nucleus and F-actin in the VSMCs cultured on the micro-grooved collagen substrate showed significant alignment in the grooves’ direction (Figure 2D).

After examination of the mechanical environment around the nucleus, thick bundles of mature stress fibers in the VSMCs on the flat collagen substrate were found to be distributed on the apical and basal surfaces of the nucleus (Figure 2C). In contrast, stress fibers in the VSMCs cultivated on micro-grooved collagen considerably concentrated in both side regions of the nucleus (Figure 2D), resulting in a significant decrease in the nucleus area, width, and volume (Figure 2E–H). These data indicated that the VSMC nuclei acquired “slim ellipsoid” morphology in the aligned cell arrangement (tissue) on the collagen micro-grooved substrate, and these alterations were strongly associated with the stress fiber distribution.

### 3.2. Cultivation on the Micro-Grooved Collagen Induces VSMC Differentiation with Proliferation Inhibition Involving YAP Signaling

**Figure 3:**
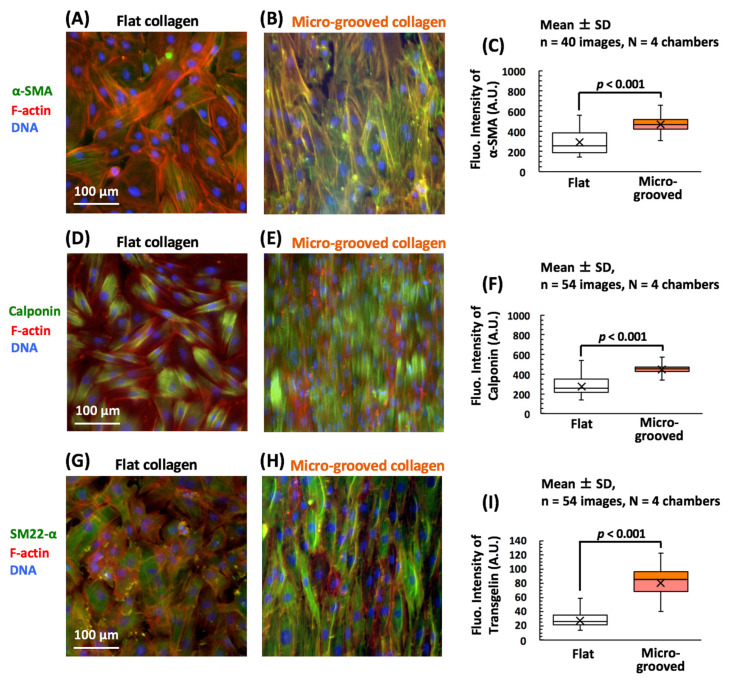
Typical examples of fluorescent images of the major marker proteins of vascular smooth muscle cells (VSMCs), e.g., α-SMA (panels **A**,**B**, green), calponin (**D**,**E**, green), and transgelin (**G**,**H**, green) in VSMCs cultured on either the flat (**A**,**D**,**G**) or micro-grooved (**B**,**E**,**H**) collagen substrate for 3 days. The actin cytoskeleton (red) and the nucleus (blue) were visualized too in all the groups. Changes in the fluorescence intensity of α-SMA, calponin, and transgelin in the VSMCs cultured on either the flat or micro-grooved collagen substrate (**C**,**F**,**I**). Fluorescence intensity/pixels were determined in each cell. The numbers (n) and numbers in parentheses (N) represent the numbers of analyzed images of the cells and the numbers of cell culture dishes (cell culture chambers), respectively. Over 500 cells were analyzed in each group.

By fluorescent image analysis, we next assessed the impact of the tissue-like cell alignment (caused by micro-grooved collagen) on VSMC differentiation (Figure 3). Fluorescence intensity levels of vascular-smooth-muscle contractility markers such as α-SMA (Figure 3A,B), calponin (Figure 3D,E), and transgelin (Figure 3G,H) were significantly higher in the aligned VSMC tissue on the micro-grooved collagen substrate, and the increase in these levels was statistically significant (Figure 3C,F,I). In particular, for transgelin, which is negatively regulated by globular actin [28], the upregulation was the most prominent, suggesting that stabilization of the filamentous structures of the actin cytoskeleton might be facilitated on the micro-grooved collagen substrate. These findings meant that the cultivation on the micro-grooved collagen substrate significantly induced VSMC differentiation with phenotypic transition (from the synthetic phenotype to contractile phenotype).

**Figure 4:**
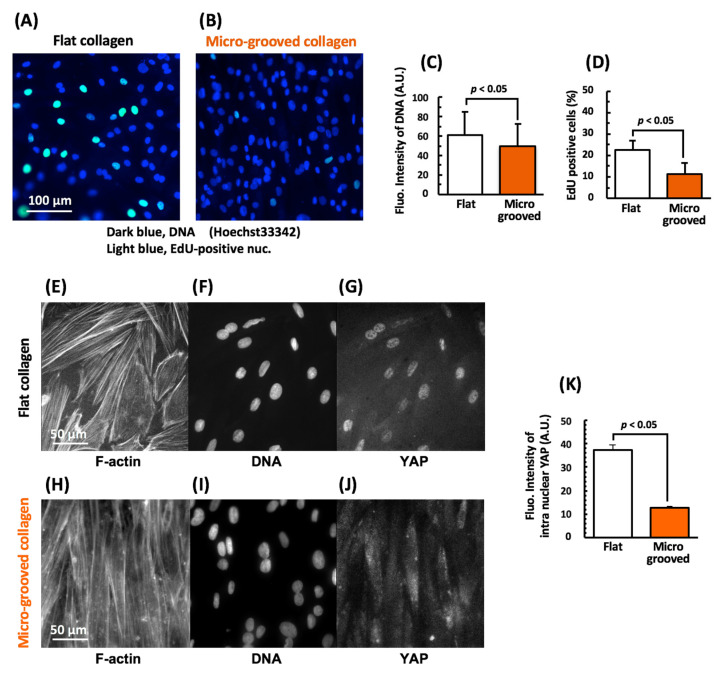
Proliferation of vascular smooth muscle cells (VSMCs), as assessed by the EdU assay. Intranuclear DNA was stained with Hoechst 33342 (**A**,**B**, dark blue) and compared with that in EdU-positive cell nuclei (**A**,**B**, Light blue). Changes in the fluorescence intensity of intranuclear DNA (**C**) and the proportion of EdU-positive cells (**D**) among the VSMCs cultured on either the flat or micro-grooved collagen substrate. Representative fluorescent images of F-actin (**E**,**H**), the nucleus (**F**,**I**), and YAP (**G**,**J**) in the VSMCs cultured on either the flat (**E**–**G**) or micro-grooved (**H**–**J**) collagen substrate for 3 days. Changes in the fluorescence intensity of intranuclear YAP (**K**). Over 100 images were analyzed in each group.

We also demonstrated that both the fluorescence intensity of Hoechst-stained DNA and the proportion of S phase cells detected by the 5-ethynyl-2′-deoxyuridine (EdU) assay were significantly lower among the VSMCs cultured on micro-grooved collagen than among those cultured on flat collagen (Figure 4A–D). Furthermore, nuclear shuttling of the Hippo effector YAP was significantly less active in the VSMCs cultured on the micro-grooved collagen substrate (Figure 4E–K). The results demonstrated that cell proliferation was significantly reduced on the micro-grooved collagen substrate that might be caused by the inhibition of nuclear shuttling of YAP (its signaling) and DNA synthesis.

### 3.3. Nuclear Mechanical Deformation Is Dramatically Smaller in Differentiated VSMCs on Micro-Grooved Collagen during Macroscopic Stretch

**Figure 5:**
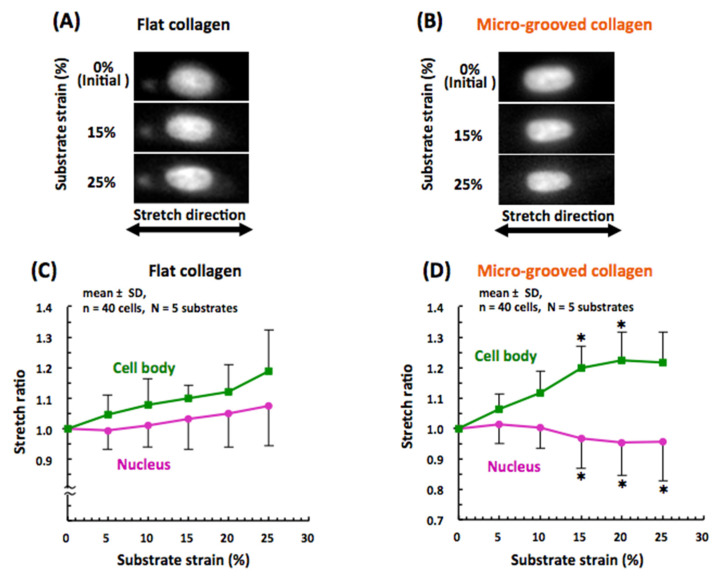
Examples of fluorescent images of a Hoechst-stained nucleus in vascular smooth muscle cells (VSMCs) cultured on either the flat (**A**) or micro-grooved (**B**) collagen substrate during macroscopic substrate stretching. The stretch ratio of the cell body and nuclear length of the VSMCs cultured on either the flat (**C**) or micro-grooved collagen (**D**) substrate; these data are plotted against the macroscopic substrate stretch. Asterisks in (**D**) indicate statistical significance compared with the cells on the flat collagen (**C**), as determined by Student’s t-test (* *p* < 0.05).

To assess changes in the nucleus–cytoskeleton interactions, we investigated stretch-induced deformation of the nucleus in the VSMCs whose contractile differentiation was facilitated by the micro-grooved collagen substrate, which enabled the modeling of the in vivo arrangement of cells in a tissue. VSMCs cultured on either flat collagen or micro-grooved collagen were subjected to simple uniaxial stretching on the stage of an inverted microscope under physiological cell culture conditions, and morphological changes of the cell body and nucleus were monitored continuously (Supplemental Videos S1,2; Figure 5A,B). The synthetic VSMCs on the flat collagen substrate gradually elongated with almost the order of magnitude of the substrate strain, and their nuclei also elongated with the substrate stretch by ~40% in comparison with the cell body (Figure 5C). By contrast, the nucleus in the differentiated VSMCs—that aligned into a tissue on the micro-grooved collagen substrate—hardly elongated during the substrate stretching, even though the cell body elongated similarly to the synthetic VSMCs on flat collagen, and the nucleus in the differentiated VSMCs showed significant shortening when the substrate strain reached 15% (Figure 5D).

The elongation of the nucleus during cell stretching was significantly depended on the intracellular F-action structures: the nuclei in VSMCs whose actin filament were completely disrupted by treatment with cytochalasin D, rarely elongated during stretching in both groups (Supplementary Figure S1).

**Figure 6:**
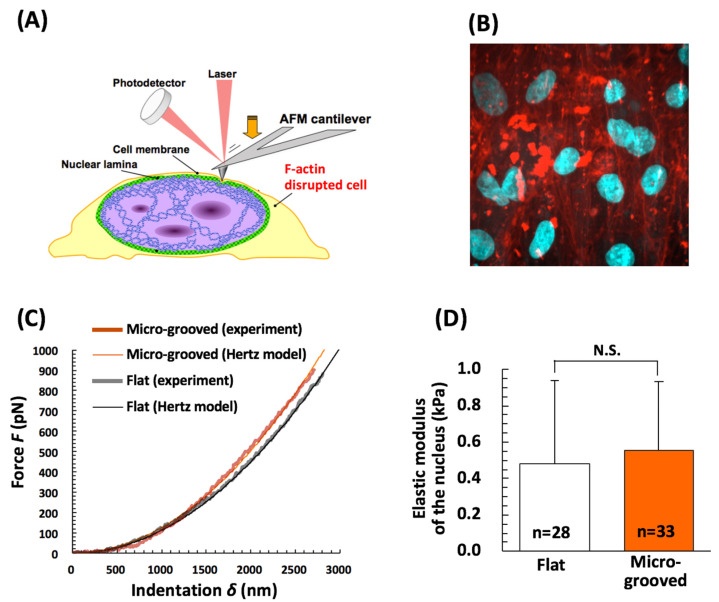
Schematic representation of the atomic force microscopy (AFM) nanoindentation test intended to measure nuclear stiffness in cells with disrupted actin filaments (**A**). An example of a fluorescent image of actin filaments and the nucleus in vascular smooth muscle cells (VSMCs) with actin filament disruption when cultured on the micro-grooved collagen substrate (**B**). Actin filaments and the nuclei were visualized using Alexa fluor-conjugated phalloidin (red) and Hoechst33342 (cyan), respectively. Representative force–indentation responses of a nucleus obtained by AFM (**C**) and elastic modulus of the nucleus region of VSMCs (**D**).

To clarify this difference in nucleus deformability during cell stretching, we quantified nuclear stiffness by AFM indentation analysis. We depolymerized actin filaments inside cells by treatment with cytochalasin D (2 µg/mL, 1 h) just before AFM indentation analyses to preclude mechanical effects of actin filaments (Figure 6A). Around the nucleus in VSMCs, actin filaments were disrupted completely by the treatment with cytochalasin D immediately before the measurements of indentations (Figure 6B). Representative force curves for the nuclei of the two cell types are depicted in Figure 6C, and the Hertzian model was found to fit the experimental data satisfactorily (R2 > 0.99). Nucleus elastic modulus of the differentiated VSMCs cultured on the micro-grooved collagen substrate (0.55 ± 0.38 kPa, n = 33) was almost the same as that of the synthetic VSMCs on the flat collagen substrate (0.48 ± 0.46 kPa, n = 28; Figure 6D), i.e., no significant difference was noted between the two groups (Figure 6D). Moreover, following actin filament disruption, the cell body region was significantly softer than the nucleus region, while no significant difference was detected between both groups (Supplementary Figure S2). Therefore, the inhibition of nucleus elongation observed in the differentiated VSMCs on micro-grooved collagen during stretching was unlikely to be due to the difference in nuclear stiffness.

**Figure 7:**
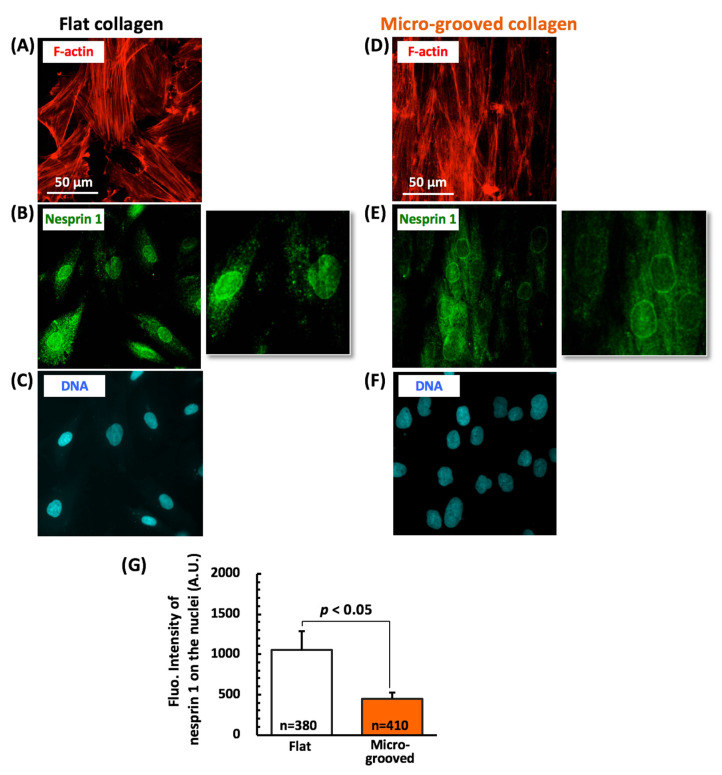
Typical fluorescent images of the actin filaments (red), nesprin 1 (green), and the nucleus (blue) in the vascular smooth muscle cells (VSMCs) cultured on either the flat (**A**–**C**) or micro-grooved (**D**–**F**) collagen substrate for 3 days. The right panels in (**B**,**E**) represent magnified images of nesprin 1 around the nuclei. Changes in the expression of nesprin 1 in the VSMCs on micro-grooved collagen (**G**). The numbers (n) represent the numbers of analyzed cells. We used 4 dishes in each group.

Hence, we finally examined the expression of nesprin 1, which tethers the actin cytoskeleton to the outer nuclear membrane (by serving as a component of the LINC complex) as another factor affecting nuclear–cytoskeletal interactions (Figure 7). The synthetic VSMCs on flat collagen featured a relatively homogeneous distribution of nesprin 1 located at the nuclear surface (Figure 7A–C). On the other hand, nesprin 1 was hardly detectable on the nuclear surface of the aligned VSMCs manifesting contractile differentiation during culturing on the micro-grooved collagen substrate (Figure 7D–F), and the expression of this protein was dramatically lower (Figure 7G).

These findings meant that the actin–nucleus connection via the nucleus–cytoskeleton linker protein was weaker in the aligned VSMC tissue whose contractile differentiation was induced by the cultivation on the micro-grooved collagen substrate, thereby causing significant inhibition of the nucleus elongation and deformation during macroscopic cell/tissue stretching.

## 4. Discussion

We fabricated a collagen micro-grooved substrate to bring about tissue-like cell alignment related to the mechanical environment of an in vivo single vascular elastic lamina. The fabrication was facile because the type I collagen matrix sheet attached to the prestretched PDMS membrane was buckled by the release of the sheet strain, thus promoting the formation of wavy-wrinkle–like micro-grooves on collagen surfaces (Figure 1). The obtained micro-grooved collagen induced elongation of cells and maintained an aligned monolayer of VSMC tissues (Figure 2B,D) in contrast to the randomly oriented multilayered cells cultivated on the conventional flat substrate (Figure 2A,C). These micro-grooves proved to be somewhat similar to the wavy structures of vascular collagen fibers in vivo [29], and micro-grooved-collagen stiffness (~370 kPa elastic modulus) is rather similar to that of an elastic lamina in vascular tissues [440 kPa elastic modulus [30]]. These observations are important for identifying adequate conditions for maintaining both mechanical and structural stability of VSMC tissue.

The tissue-like alignment of VSMCs on micro-grooved collagen also induced F-actin remodeling: the mature stress fibers distributed in the apical part of the cells almost disappeared and became concentrated in the cells’ both side regions consisting of cell–cell junctions (Figure 2D). Consequently, the nucleus underwent dramatic morphological changes to adopt a slenderer shape with a volume reduction (Figure 2E–H), which led to a decrease in the fluorescence intensity of Hoechst-stained DNA (Figure 4C) and inhibition of DNA synthesis (Figure 4D) and of cell proliferation.

Furthermore, YAP nuclear localization turned out to be significantly inhibited in the aligned VSMC tissue on the micro-grooved collagen substrate (Figure 4J,K). Transcriptional coactivators YAP and TAZ recently became known as major mediators of the mechanotransduction that regulates multiple cellular processes, including cell proliferation. The inhibition of YAP nuclear localization is also observed after ECM softening [31] and contact inhibition of cell proliferation [32], which are deeply involved in actomyosin inactivation [32,33]. Thus, in this study, the tissue formation in aligned cells with F-actin remodeling during culturing on micro-grooved collagen probably reduced mechanical tension for VSMCs, thereby resulting in YAP cytoplasmic retention, which inhibits cell proliferation.

Levels of contractile proteins’ expression were significantly greater in the aligned VSMC tissue on the collagen micro-grooved substrate, implying that VSMC differentiation was more active on the micro-grooved collagen (Figure 3). The most prominent upregulation of transgelin along with the F-actin reorganization (its relocation from the apical cell surface to both side regions of the cells) indicates that F-actin stabilization was promoted in the region of the cell–cell contact during the cultivation on the micro-grooved collagen substrate. Some reports have suggested that actin stress fibers situated at the nucleus apical surface have more dynamic structures and generate greater internal mechanical forces than do other F-actin structures in cells cultured on a two-dimensional (2D) matrix [34,35]. Moreover, it was recently demonstrated that suppressing YAP/TAZ expression attenuates the stretch-induced dedifferentiation of VSMCs from the contractile to synthetic phenotype [36]. Accordingly, in this study, the F-actin remodeling (i.e., its relocation from the apical cell surface to both sides of the cell) during the tissue formation in aligned VSMCs on micro-grooved collagen may have facilitated intracellular force reduction and consequently inhibited YAP nuclear localization and improved VSMC contractile differentiation.

The finding of cell deformation under macroscopic stretching revealed that the synthetic VSMCs featuring mature stress fibers around the nucleus on the conventional flat collagen closely follow the stretch deformation imposed by the substrate, and their nucleus also deforms significantly under the applied substrate stretch, although the deformation value of the nucleus was ~40% of that of the cell body (Figure 5A,C). This result represents a clear mechanical coupling between the ECM and nucleus in VSMCs on the flat collagen substrate and is in good agreement with the nucleus deformation in adherent cells (under 2D culture conditions) reported in other studies [37,38]. Notably, the differentiated VSMCs manifesting tissue formation in aligned cells on micro-grooved collagen behaved quite differently; their cell body extended with substrate strain, while their nucleus rarely elongated after the stretching (Figure 5B,D), even though their nuclear stiffness did not change significantly (Figure 6). The nucleus of the aligned VSMCs sometimes showed their shortening when the substrate strain reached over ~15% (Figure 5D), which represents the threshold value of physiological strain in vascular tissue in vivo [18]. Furthermore, the expression of nesprin 1 (constituent of LINC complex) at the apical surface of the nucleus was significantly lower in the aligned VSMCs on micro-grooved collagen (Figure 7). One report shows that the disruption of the actin–nucleus connection maintained by the LINC complex reduces nucleus deformation per unit of imposed cell strain by approximately 50% [39]. A loss of the actin–nucleus connection mediated by the LINC complex may prevent the activation of cytoskeleton- and nucleus-associated mechanosensitive factors, including YAP signaling, in response to a stretch [40]. Consequently, such a loss of the actin–nucleus connection induced by the aligned VSMC arrangement on micro-grooved collagen dramatically inhibits the nucleus deformation during physiological stretching. This type of inhibition of nucleus deformation might have important functions not only in the maintenance of the VSMC contractile phenotype but also in the protection of biomechanical and physiological integrity of the cell nucleus from external mechanical disturbances in vivo.

## Data Availability

Not applicable.

## Supplementary Materials

The following are available online at https://www.mdpi.com/article/10.3390/bioengineering8090124/s1, Video S1: VSMC tissue on the flat collagen substrate during uniaxial stretching, Video S2: The aligned VSMC tissue on the micro-grooved collagen substrate during uniaxial stretching. Figure S1: Examples of fluorescent images of a Hoechst-stained nucleus in the actin filament-disrupted vascular smooth muscle cells cultured on either the flat (A) or micro-grooved (B) collagen substrate during macroscopic substrate stretching. The actin filaments of the cells were completely disrupted by cytochalasin D (2 µg/mL, 1 h) just before stretching test. The stretch ratio of the cell body and nuclear length of the cells cultured on either the flat (C) or micro-grooved collagen (D) substrate. Figure S2: AFM force–indentation responses of nonnuclear region (cell body) of VSMCs following actin filament disruption with cytochalasin D (2 µg/mL, 1 h) (A) and elastic modulus of the cell body region (D). No significant difference was detected between in the cells cultured on the flat and micro-grooved collagen substrates.
