# Supplementary material for: A Loss of Nuclear—Cytoskeletal Interactions in Vascular Smooth Muscle Cell Differentiation Induced by a Micro-Grooved Collagen Substrate Enabling the Modeling of an In Vivo Cell Arrangement
Source: Bioengineering (Basel). 2021 Sep 12;8(9):124. doi: 10.3390/bioengineering8090124 (PMC8470899; doi:10.3390/bioengineering8090124)
Supplement: Supplementary file 1 [file bioengineering-08-00124-s001.zip › supplementary material/MDPI_Bioengineering2021_Nagayama_SuppleFigs.pdf]

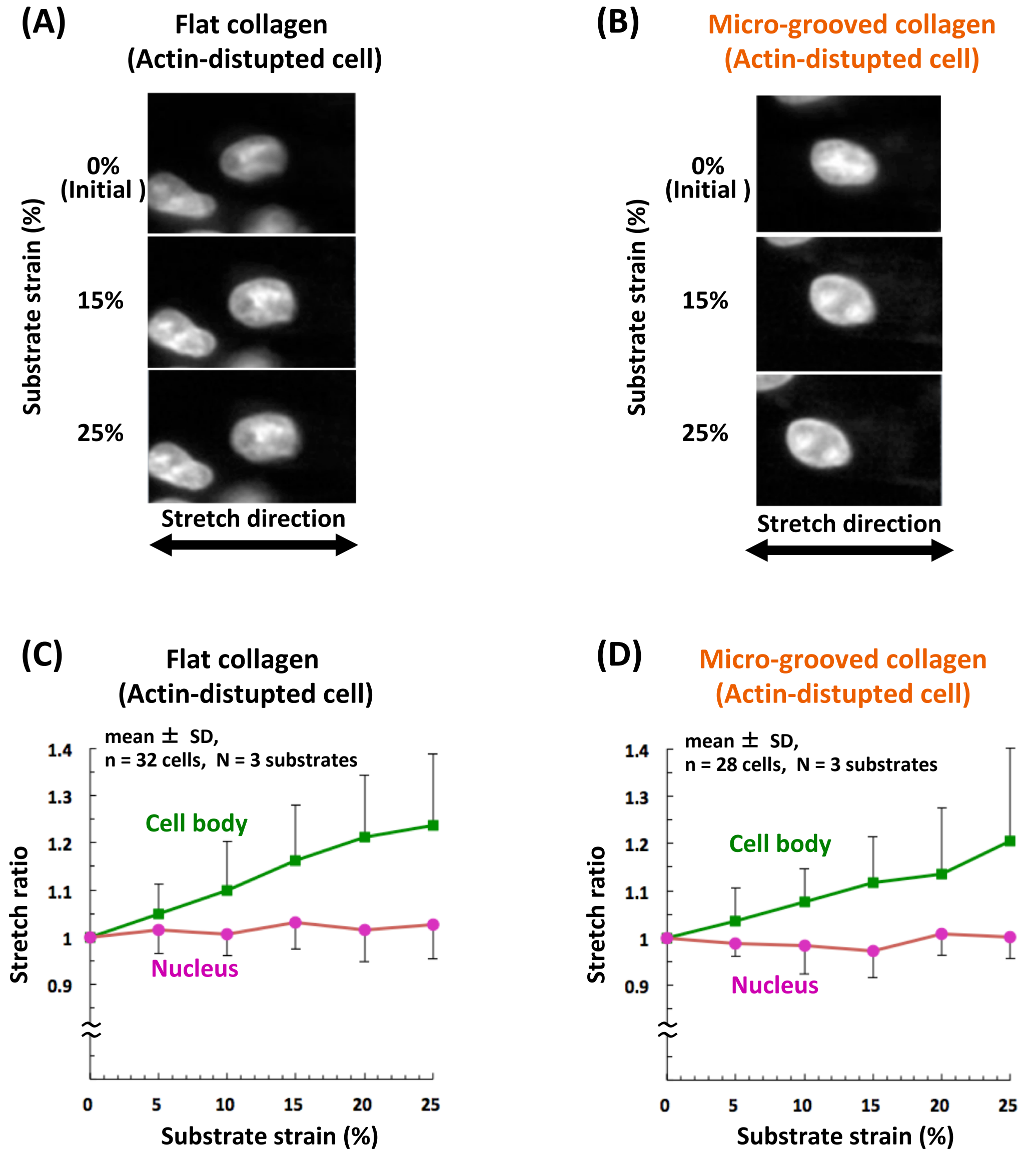

Figure S1: Examples of fluorescent images of a Hoechst-stained nucleus in the actin filament-disrupted vascular smooth muscle cells cultured on either the flat (A) or micro-grooved (B) collagen substrate during macroscopic substrate stretching. The actin filaments of the cells were completely disrupted by cytochalasin D (2 μg/mL, 1 h) just before stretching test. The stretch ratio of the cell body and nuclear length of the cells cultured on either the flat (C) or micro-grooved collagen (D) substrate.

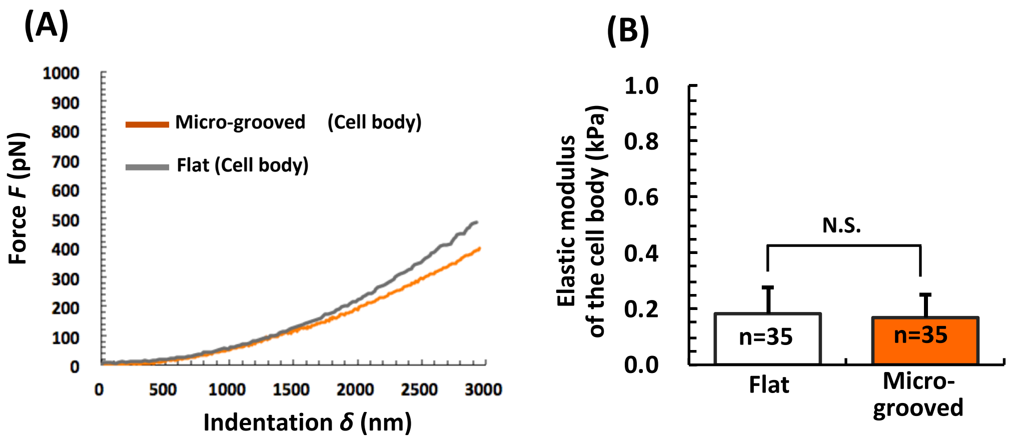

Figure S2: AFM force–indentation responses of nonnuclear region (cell body) of VSMCs following actin filament disruption with cytochalasin D (2 μg/mL, 1 h) (A) and elastic modulus of the cell body region (D). No significant difference was detected between in the cells cultured on the flat and micro-grooved collagen substrates.
